# BeCaked: An Explainable Artificial Intelligence Model for COVID-19 Forecasting

**DOI:** 10.1038/s41598-022-11693-9

**Published:** 2022-05-13

**Authors:** Duc Q. Nguyen, Nghia Q. Vo, Thinh T. Nguyen, Khuong Nguyen-An, Quang H. Nguyen, Dang N. Tran, Tho T. Quan

**Affiliations:** 1 Faculty of Computer Science and Engineering, Ho Chi Minh City University of Technology (HCMUT), Ho Chi Minh City, Vietnam; 2 School of Medicine, Ho Chi Minh City, Vietnam; 3 Vietnam National University Ho Chi Minh City, Ho Chi Minh City, Vietnam; 4 Faculty of Public Health, University of Medicine and Pharmacy at Ho Chi Minh City, Ho Chi Minh City, Vietnam

**Keywords:** Bioinformatics, Computational models, Machine learning, Disease model, Computer science, Differential equations, Dynamical systems, Time series, Applied mathematics, Computational science, Computer science, Public health

## Abstract

From the end of 2019, one of the most serious and largest spread pandemics occurred in Wuhan (China) named *Coronavirus* (COVID-19). As reported by the World Health Organization, there are currently more than 100 million infectious cases with an average mortality rate of about five percent all over the world. To avoid serious consequences on people’s lives and the economy, policies and actions need to be suitably made in time. To do that, the authorities need to know the future trend in the development process of this pandemic. This is the reason why forecasting models play an important role in controlling the pandemic situation. However, the behavior of this pandemic is extremely complicated and difficult to be analyzed, so that an effective model is not only considered on accurate forecasting results but also the explainable capability for human experts to take action pro-actively. With the recent advancement of *Artificial Intelligence* (AI) techniques, the emerging *Deep Learning* (DL) models have been proving highly effective when forecasting this pandemic future from the huge historical data. However, the main weakness of DL models is lacking the explanation capabilities. To overcome this limitation, we introduce a novel combination of the *Susceptible-Infectious-Recovered-Deceased* (SIRD) compartmental model and *Variational Autoencoder* (VAE) neural network known as BeCaked. With pandemic data provided by the Johns Hopkins University Center for Systems Science and Engineering, our model achieves 0.98 $R^2$ and 0.012 *MAPE* at world level with 31-step forecast and up to 0.99 $R^2$ and 0.0026 *MAPE* at country level with 15-step forecast on predicting daily infectious cases. Not only enjoying high accuracy, but BeCaked also offers useful justifications for its results based on the parameters of the SIRD model. Therefore, BeCaked can be used as a reference for authorities or medical experts to make on time right decisions.

## Introduction

*Deep Learning* (DL)^1^, a subarea of machine learning, has been applied in many tasks such as speech recognition, object detection, natural language processing, etc. with noticeably high accuracy. Due to its powerful computation capability, DL models are proven highly effective once handling huge datasets whose volumes easily make human beings overwhelming.

Thus, as the pandemic of *Coronavirus* (COVID-19)^2,3^ has been spreading on a worldwide scale and posing a serious threat to daily life of humanity, DL is considered as an effective machine learning approach to analyze the massive dataset of patient records of infected and tested cases, which can be collected on the daily basis and presented as a *sequence* of historical data. Due to its data-driven learning mechanism, DL-based approaches usually introduce highly accurate rates when predicting the increase of infectious epidemics from the past historical data. In particular, a special kind of Deep Learning known as *Recurrent Neural Network* (RNN)^4^ and its advanced version, *Long Short Term Memory* (LSTM)^5^, enjoy visibly better performance once compared to traditional methods such as ARIMA, SEIR, etc.^6–8^ and deliver significant results for some countries, for instance, Canada^9^ and European countries^10^. It is because the operational mechanism of this network kind is effectively suitable to process sequence data.

**Figure 1:**
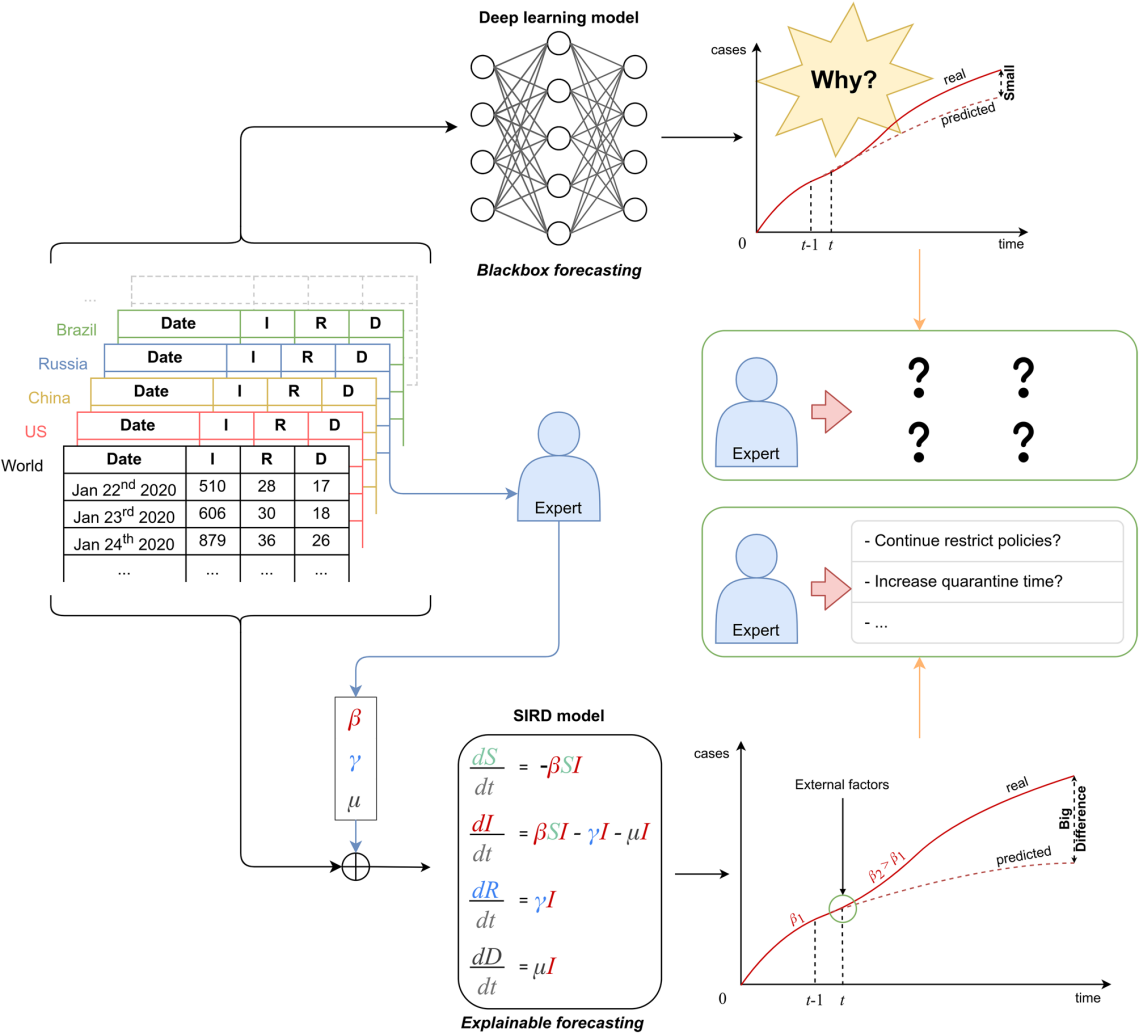
Illustration of the usage of deep learning and explainable model for COVID-19 forecast.

Nevertheless, the contribution of DL-based methods is limited to the fact that their results are often given in a black-box manner, making them unexplainable in terms of the internal properties of the pandemic. Thus, they hardly provide experts with explicit declarative knowledge, based on which a corresponding action plan can be prepared. For instance, let us consider some motivating situations given in Fig. 1. Due to a very large number of patient records rapidly collected and processed, a well-trained Deep Learning model can predict a certain increase of infectious cases in the next few days. However, this model generally could not explain for itself the reason behind such a trend. Hence, medical experts suffer difficulty from retrieving the root cause of the situation and proposing proper actions to improve the status. In the other words, the problem which Deep Learning as well as many machine learning models are facing is that predicted output are not accompanied by a justification or anything that substantiates insights on what the models have learned. To solve this problem, a new generation of machine learning models known as *Explainable Artificial Intelligence* (Explainable AI)^11^ has emerged and is expected to overcome the weaknesses of the black-box machine learning models. This generation not only makes machine learning models more explainable, while still maintaining a high level of learning performance, but also enables us to trust, understand and productively manage the emerging development of AI systems.

In terms of an epidemic, there have been many studies on modeling and forecasting its future. Technically, we can divide those models into two groups including *mathematical models* and *machine learning models*^12^. Most mathematical models are based on a well-known compartmental model introduced by Kermack and McKendrick in 1927^13^. However, the parameters of those models are biasedly determined; thus, they are subjective and unobvious. In this paper, a model called *Susceptible–Infectious–Recovered–Deceased* (SIRD)^14,15^ which is one of the most commonly used mathematical models in the past to calculate epidemic outbreaks, is considered. It is illustrated in Fig. 1, when successfully observing the parameters of the SIRD model from the recorded cases, experts can better understand the situation and suggest suitable actions. For example, upon witnessing that the infectious rate is significantly reduced while the deceased rate is relatively high, one can conclude that even though the reported number of infectious cases is still seriously high, the threat of infection in communities is now under control and suggest to lift the lock-down restriction in some certain regions.

Even though mathematical models can give useful hints for human experts from their internal parameters, to estimate such parameters from vast sources of real historical data is by no means a trivial task, which can be potentially handled by DL models. Hence, the combination of Deep Learning models and the mathematical SIRD model interestingly promises an Explainable AI solution to deal with the terrifying COVID-19 pandemic. In this paper, we propose a semi-supervised model known as *BeCaked* (Be Careful and Keep Distance) to realize this vision. Our work is inspired by the *Variational-LSTM Autoencoder* model^16^ where the neural architecture of *Autoencoder* (AE)^17^ is combined with the previously discussed LSTM to encode the data produced from LSTM into a higher informative representation for better processing. However, the results from this model are still unexplainable. To address this, we modify the Autoencoder architecture to enforce it to encode the processed sequence data produced by the LSTM layers into SIRD model parameters. Thus, the forecasting results of this end-to-end trainable architecture can be comprehensible for human experts in terms of those SIRD parameters, making our AI approach explainable. Moreover, the model makes use of the advantages of semi-supervised learning algorithms (the Autoencoder network specific), which do not need labeled data accompanied by sophisticated loss functions.

The rest of this paper is organized as follows. Section “Related works” highlights some related works including classical mathematical and modern machine learning models. In “Preliminaries”, we recall background knowledge on the Deep Learning models of LSTM and *Variational Autoencoder* (VAE). The famous SIRD model and its capability of explainability are presented in “The SIRD model and its explainability”. Next, the technical details of the BeCaked model are presented in “The BeCaked model”. In “Performance evaluation”, insightful experiments are conducted with real COVID-19 data. Those experiments also show that our explainable BeCaked not only enjoys high accuracy of prediction, as compared to some *state-of-the-art* (SOTA) models, but can also analyze what is going on with the pandemic, illustrated by real data from some major countries in the world. We discuss our model and its achievements in “Discussion”. Finally, “Conclusion” concludes our study and gives some future possible improvements. We also attach an appendix about the name “BeCaked” (“Appendix 1”) and illustrations of web-based system which we have deployed our BeCaked model on (“Appendix 2”) for interested readers.

## Related works

Since the COVID-19 pandemic began to spread, there has been a lot of research to solve the problem of sequencing the virus gene, finding a cure, a vaccine, predicting the effect and extent of transmission spread of the pandemic, etc. To be honest, we cannot deny the benefits of pandemic forecasting models. Thanks to them, countries can detect infected people early and slow down the spread of this pandemic. Since then, medical researchers have more time to research and find vaccines and medicines. In this section, we analyze the advantages and disadvantages of the latest mathematical and Deep Learning models to predict this pandemic and compare them with the model we have proposed.

### Mathematical models

Most of the mathematical models currently used for epidemic prediction are developed based on the *Susceptible-Infectious-Recovered* (SIR) model of Kermack and McKendrick^13^. The common point of these mathematical prediction models is the reliability and the predictable results. By converting factors that influence the epidemic into differential equations and integrating them with existing equations, researchers have created more variations with more realistic predictability than the original one and suitable for many types of epidemic. Some highlighted recent research can be listed as follows: SEIRD^7^, SIRD^15^, SEIPEHRF^18^, etc. The main weakness of these mathematical models is that they require transition rates between states and those numbers are not easy to estimate accurately. Because the experts estimating those rates are still human, so their predictions still contain “humanity” and sometimes do not have enough “sensitivity”. Therefore, the performance of those models is often not as high as they were expected.

Some other models that can be used to forecast this pandemic are regression-based models. These models depend on both their hyperparameters and the historical data. As a consequence, their performances are almost the same as the SIR-based model. Some well-known models can be listed such as *Geographically Weighted Regression* (GWR)^19^, ARIMA^10^ and its extensions.

### Machine learning models

Towards machine learning models for forecasting, the very first thing to be mentioned is that they achieve high performance when being applied in this COVID-19 pandemic. There are so many models, varying from simple to complex in their architecture. Recent studies notice that they can assemble some external factors of the pandemic to make the prediction more accurate. We can consider some outstanding ones such as LSTM-based models^7,10^, Variational-LSTM Autoencoder^16^, NARNN^10^, etc.

Apart from forecasting from time series data, other multimedia data, e.g. X-ray images, are also incorporated into the latest Deep Learning models, mostly for diagnosis purposes. In recent years, various works^20–22^ have been reported on hybrid approaches that fuse features extracted from X-ray images into Deep Learning models for medical diagnosis. Besides, there is also a benchmarking work of Mohammed et al.^23^ which is made for selecting the best model using information theory.

In general, diagnosis models using multimodal approaches have achieved some remarkable results, and we can see that some of their results have already been applied to real medical practices. However, in order to effectively respond to the pandemic, forecasting models are still highly demanded. As discussed, Deep Learning models have demonstrated high accuracy in terms of performance. However, as a trade-off, they are extremely complex and need more detailed input data (such as the contact information, the number of testing or quarantines, etc.), which will result in an unsuitable situation when using those SOTA models in developing countries where modern technology is not reachable^24^. Also, besides their good performance, the only things we get from those models are the number of cases. They can not give us insights into how they predict those values. Therefore, although their forecasting performances are usually high, they could not convince epidemiologists about their reliability. Because the pandemic situation changes every hour, every day, those models can predict well at this moment, but no guarantee that they will do the same for further moments. Moreover, experts need more information than the only number of cases to control the pandemic, so we can easily consider that almost machine learning models could not satisfy them.

Our proposed BeCaked model also takes advantage of the LSTM layer when using it to extract “sequence” features of historical time-series data. As a result, our model can find the relationship between the difference in the number of considered cases in the previous days and the parameters ($\beta$, $\gamma$, $\mu$) of the SIRD model. This is the same as for the above LSTM-based models that successfully find the relationship between the number of cases in the past and the future. While those models can not elucidate clearly their forecasting results, our BeCaked one represents the forecasting by explanations based on the connection of ($\beta$, $\gamma$, $\mu$) and cases. Therefore, our model has not only high precision but also denotes the reason why its predictions are like that.

## Preliminaries

### LSTM neural network

**Figure 2:**
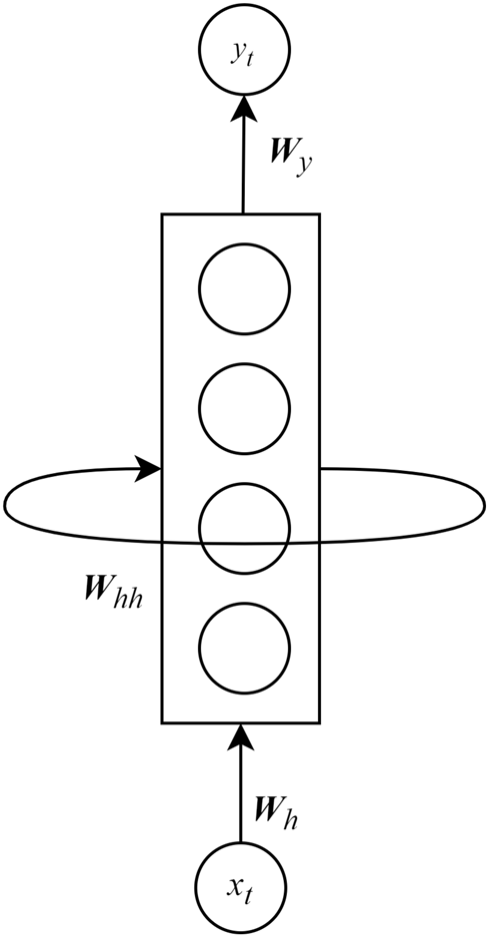
The physical architecture of an RNN.

**Figure 3:**
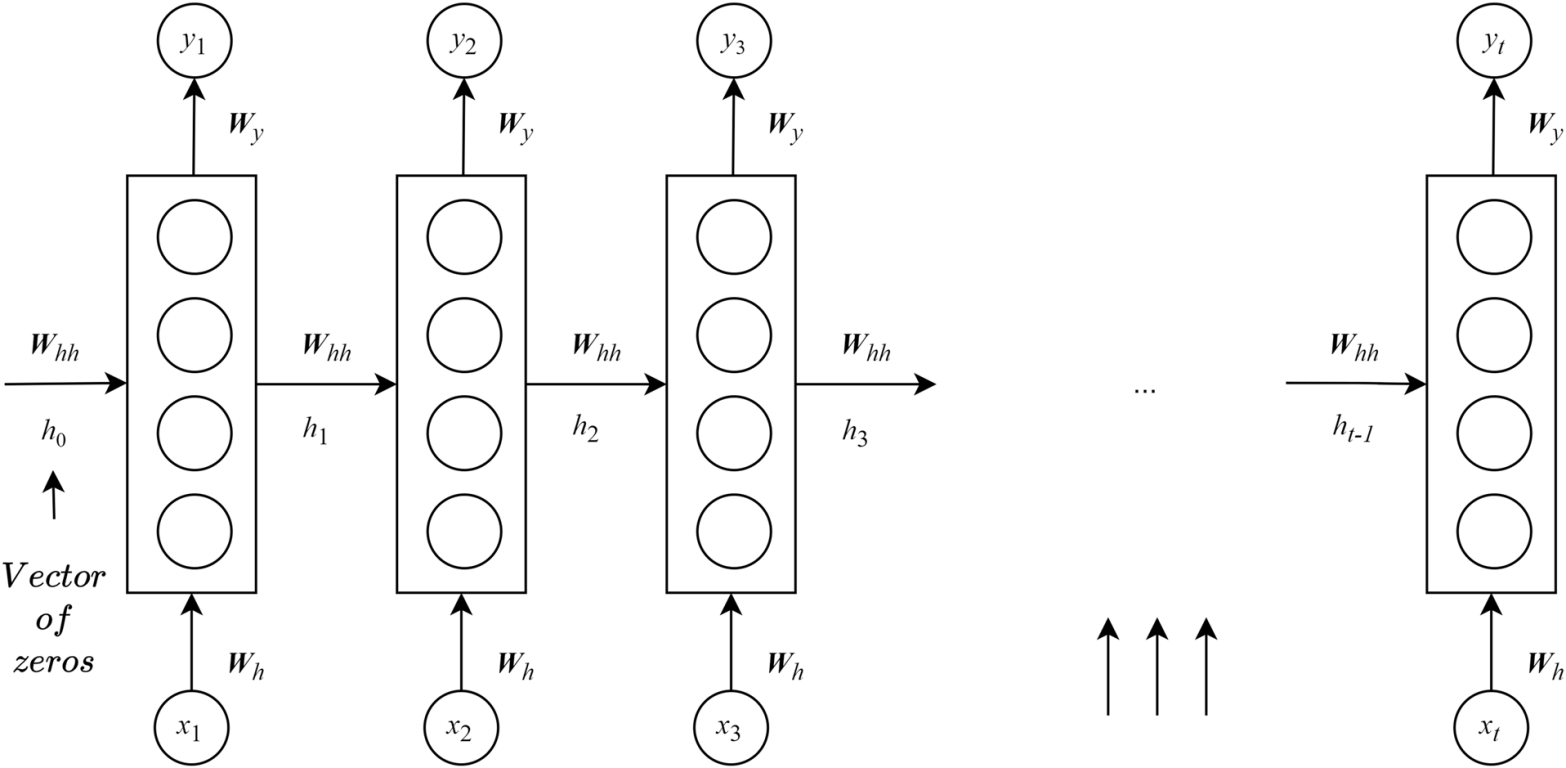
An unfolded RNN.

Modeling time-series data is likely impossible when using the standard *Multilayer Perceptron* (MLP)^4^ due to a lack of correlations between them. Therefore, an *Recurrent Neural Network* (RNN) was developed in the 1986 by Rumelhart^4^ and improved by Werbos^25^ and Elman^26^ for addressing that type of problem. In general, the construction of an RNN is similar to *Feed-forward Neural Network* (FNN)^27^ with the distinction that a presence of connections between hidden layers is spanned through adjacent time steps. By these connections, an RNN can retain the properties of information because of the share-weighted characteristic, providing an ability to learn temporal correlations with high accuracy even when the locations of featured events are likely far away from each other. Figure 2 presents the basic architecture of an RNN, which has physically one layer. At the time *t*, this network will produce output $y_t$ from the input $x_t$. However, the output of this network at the previous iteration will also be used as a part of the input of the next step, or *recurrent input*, together with new actual input. Similar to a typical MLP, RNN uses some layers of perceptron to learn suitable weights in the backpropagation manner when processing input, output and recurrent input, denoted as ${{{\varvec{W}}}}_{h}$, ${{{\varvec{W}}}}_{y}$ and ${{{\varvec{W}}}}_{hh}$, respectively^4^. Thus, when handling a sequence of data $x_t$, an RNN can be logically unfolded as a recurrent multilayer network, as depicted in Fig. 3. The whole process from getting input to producing output in RNN is expressed in Eq. (1a, 1b).

1a $$\begin{aligned} h_t= &\, {} \delta ({{{\varvec{W}}}}_{h}x_t + {{{\varvec{W}}}}_{hh}h_{t-1} + {{{\varvec{b}}}}_h) \end{aligned}$$

1b $$\begin{aligned} y_t= &\, {} \zeta ({{{\varvec{W}}}}_{y}h_t + {{{\varvec{b}}}}_y) \end{aligned}$$

In (1a, 1b), $h_t$ is the hidden state of RNN at the time *t*; ${{{\varvec{W}}}}_{h}$, ${{{\varvec{W}}}}_{hh}$ and ${{{\varvec{W}}}}_{y}$ are learnable weight matrixes for input-to-hidden, hidden-to-hidden, and hidden-to-output connections, respectively; ${{{\varvec{b}}}}_h$ and ${{{\varvec{b}}}}_y$ are bias coefficients; $\delta$ and $\zeta$ are non-linear activation functions which can be chosen based on a specific problem.

Even though the RNN is theoretically a simple and powerful model, it is difficult to learn properly due to a limit in learning long-term dependencies, caused by two well-known issues in training a model which are *vanishing* and *exploding gradient*^28^. The vanishing gradient will become worse when a *sigmoid*^4^ activation function is used, whereas a *Rectified Linear Unit* (ReLU) can easily lead to an exploding gradient. Fortunately, a formal thorough mathematical explanation of the vanishing and exploding gradient problems was represented by Bengio^29^, analyzing conditions under which these problems may appear.

**Figure 4:**
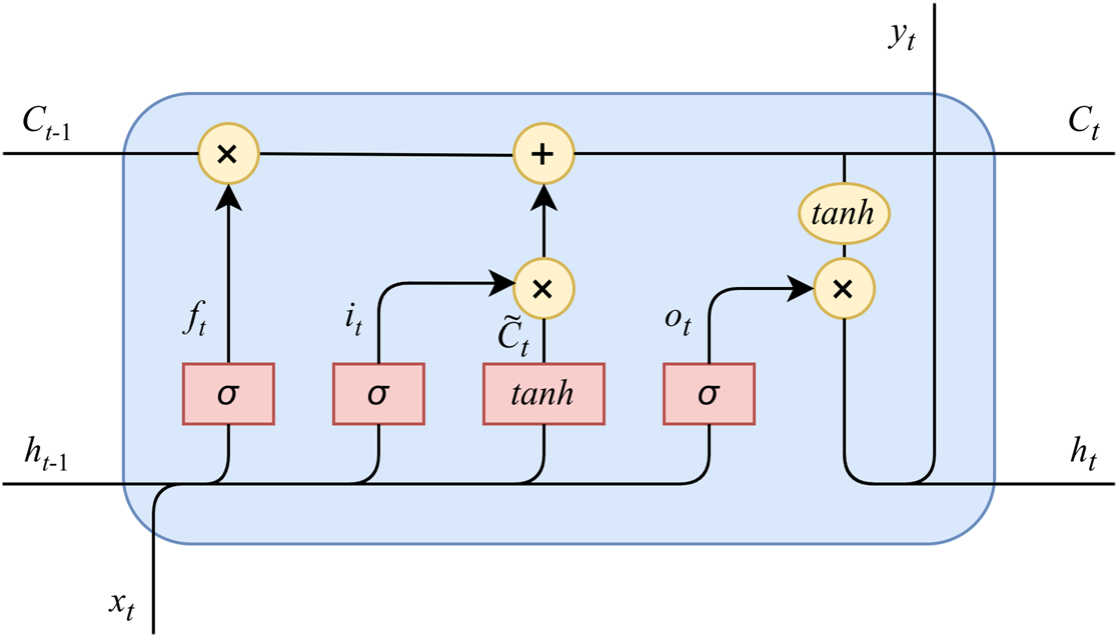
The structure of an LSTM cell.

To deal with the long-term dependency problem, a developed version of RNN was introduced by Hochreiter and Schmidhuber in 1997, called *Long Short Term Memory* (LSTM)^5^. LSTM has overcome the limitations of RNN and delivers a higher performance by using a hidden layer as a memory cell instead of a recurrent cell (see Fig. 4). In the standard LSTM model, processing information is more complicated when modules containing computational blocks are repeated over many timesteps to selectively interact with each other to determine which information will be added or removed. This process is controlled by three gates namely *input gate*, *output gate*, and *forget gate*. Controlling the flow of information inside an LSTM model is calculated using Eqs. (2a)–(2f).

2a $$\begin{aligned} i_t= &\, {} \sigma ({{{\varvec{W}}}}_{i}x_{t} + {{{\varvec{W}}}}_{hi}h_{t-1} + {{{\varvec{b}}}}_{i})\end{aligned}$$

2b $$\begin{aligned} f_t= &\, {} \sigma ({{{\varvec{W}}}}_{f}x_{t} + {{{\varvec{W}}}}_{hf}h_{t-1} + {{{\varvec{b}}}}_{f})\end{aligned}$$

2c $$\begin{aligned} o_t= &\, {} \sigma ({{{\varvec{W}}}}_{o}x_{t} + {{{\varvec{W}}}}_{ho}h_{t-1} + {{{\varvec{b}}}}_{o})\end{aligned}$$

2d $$\begin{aligned} {\tilde{C}}_t= &\, {} tanh({{{\varvec{W}}}}_{C}x_{t} + {{{\varvec{W}}}}_{hC}h_{t-1} + {{{\varvec{b}}}}_{C})\end{aligned}$$

2e $$\begin{aligned} C_{t}= &\, {} f_t \otimes C_{t-1} + i_t \otimes {\tilde{C}}_t\end{aligned}$$

2f $$\begin{aligned} h_{t}= &\, {} o_t \otimes tanh(C_{t-1}) \end{aligned}$$

In Eqs. (2a)–(2f), $i_t$, $f_t$, $o_t$, $C_t$, $h_t$ denote input gate, forget gate, output gate, internal state, and hidden layer at the time *t* respectively. Here, ${{{\varvec{W}}}}_i$, ${{{\varvec{W}}}}_f$, ${{{\varvec{W}}}}_o$, ${{{\varvec{W}}}}_C$, and ${{{\varvec{W}}}}_{hi}$, ${{{\varvec{W}}}}_{hf}$, ${{{\varvec{W}}}}_{ho}$, ${{{\varvec{W}}}}_{hC}$ and ${{{\varvec{b}}}}_i$, ${{{\varvec{b}}}}_f$, ${{{\varvec{b}}}}_o$, ${{{\varvec{b}}}}_C$ represent the weight matrixes and biases of three gates and a memory cell, in the order given. Concretely, the activation function, *sigmoid* ($\sigma$), helps an LSTM model control the flow of information because the range of this activation function varies from zero to one so if the value is zero, all of the information is cut off, otherwise, the entire flow of information passes through. Similarly, the output gate allows information to be revealed appropriately due to the *sigmoid* activation function then the weights are updated by the element-wise multiplication of output gate and internal state activated by non-linearity *tanh* function. With the pivotal component which is the memory cell accommodating three gates: input, forget, and output gate, LSTM has overcome limitations of RNN, enhancing the ability to remember values over an arbitrary time interval by regulating the flow of information inside the memory cell. Therefore, LSTM possesses a capacity to work tremendously well on learning features from sequential data such as documents, connected handwriting, speech processing, or anomaly detection, etc.^30^.

### Autoencoder and variational autoencoder

**Figure 5:**
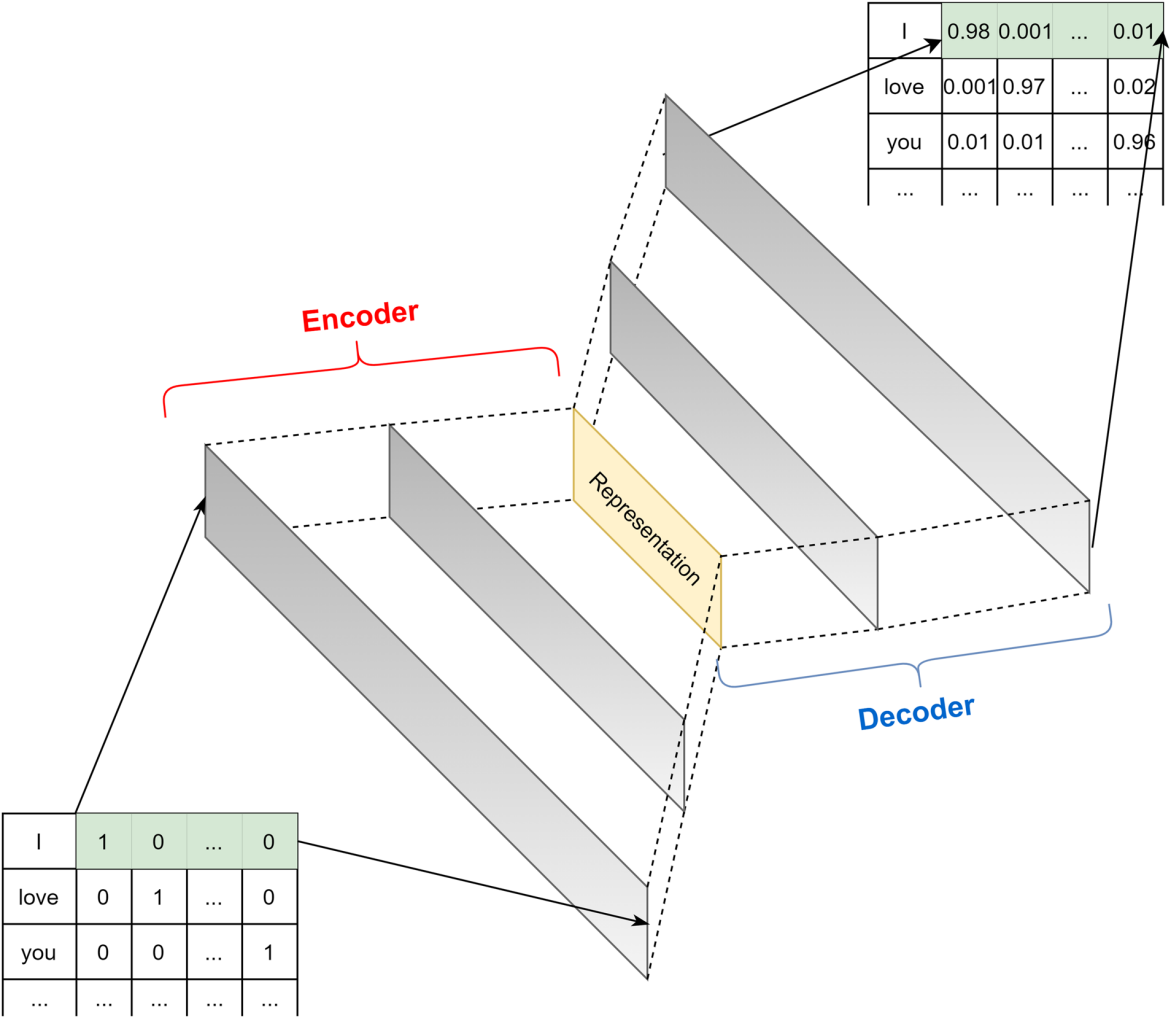
The concept of autoencoder.

*Autoencoder* (AE)^17^ is a type of neural network designed to attempt to copy its input to its output, concurrently producing an *encoding representation* of the input. The network can be described as a construction of two parts, and the internal process can be observed in Fig. 5.

- *Encoder*: this part of the neural network will compress the input into a latent-space representation which can be represented as an encoding function A: $f: X \rightarrow H$
- *Decoder*: this part, in contrast to the encoder, try to reconstruct the input from the latent-space representation which can be described as a reconstruction function B: $g: H \rightarrow R$ where the distance between *R* and *X* needs to be minimized.

Therefore, Autoencoder can be described as an unsupervised learning process that the bottleneck hidden layer will force the network to learn from a latent space representation, resulted from automatically encoding the input data, whereas, the reconstruction loss will make the latent representation contain as much information of the input as possible. This learning method enables machines to capture the most meaningful features which accurately represent the input and ignore others that do not really describe the input data.

Perhaps the most popular usage of Autoencoder is *encoding*. As its name implies, after being properly trained, the encoder can be used to encode any input to the corresponding latent-space representation. Autoencoder has been deployed in various fields of AI. In the area of *Natural Language Processing* (NLP), the Autoencoder network is widely used as a basic method for word embedding or machine translation tasks. Also, it has proved its power to solve some problems in *Computer Vision* (CV) such as image compressing^31^, image denoising^32^, etc. Recently, the Autoencoder network has been developed with many improvements in order to fit more problem types. With its flexible transformation ability, the Autoencoder can be changed its training method for other problems (such as *Variational Autoencoder* (VAE)^33^ for Recommender system^19^), or its architecture such as adding or removing its hidden layers with more specific ones like *Convolutional Neural Network* (CNN)^34^, LSTM or itself (such as Autoencoder in Autoencoder for data representation^35^).

In the standard Autoencoder network, the encoder and the decoder are usually implemented as *Fully-connected* (FC) or CNN. Therefore, the input in Autoencoder is encoded into latent deterministic variables. Whereas, its attention to VAE generates a probabilistic distribution over latent random variables by using Bayes’s rule to approximate the probability *p*(*code*|*input*) with the presence of the mean $\mu$ and standard deviation $\sigma$. Reversely, the decoder, inversely approximating the probability *p*(*output*|*code*), will be a scaffolding for the encoder to learn the rich representations of data^36^. In the original VAE model, the encoder is used to learn the parameters of data distribution from the input space. This architecture can be adapted to learn other kinds of distribution parameters such as the one used in aspect-based opinion summary^37^, which extends the VAE model to learn the parameters of Dirichlet distributions in the problem of topic modeling. In this work, we combine VAE with LSTM to learn the parameters of the SIRD model which will be discussed in the next section.

## The SIRD model and its explainability

**Figure 6:**
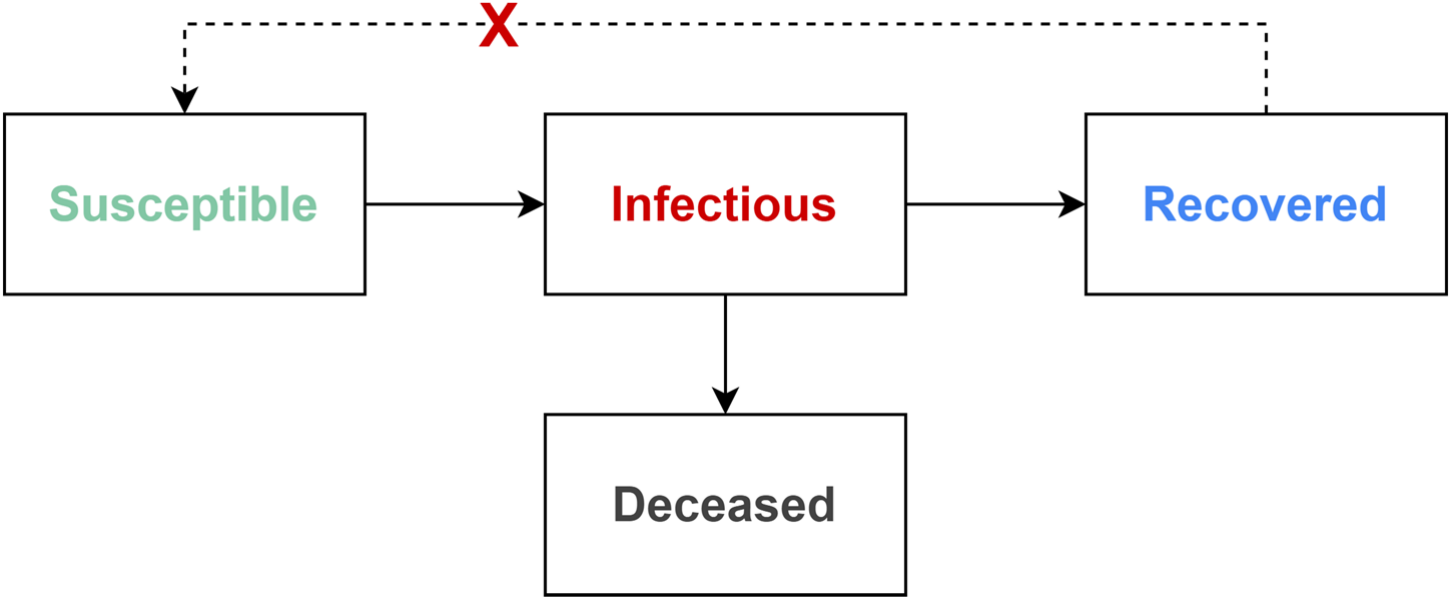
The concept of the SIRD model.

The *Susceptible-Infectious-Recovered-Deceased* (SIRD) model is one of the most commonly used in the past to describe epidemic outbreaks^38–40^. The model demonstrates four states known as *Susceptible*, *Infectious*, *Recovered* and *Deceased* of people in a population isolated under the spread of an infectious epidemic. In most infectious epidemic, the simple SIRD model assumes that infected people will be immune from that epidemic if they have recovered^41^. Figure 6 presents the transitions between states in the SIRD model. In detail, people in *Susceptible* state move to *Infectious* state if they are infected by another one. When a person is in *Infectious* state, he can be cured successfully and then moves to *Recovered* state or unluckily moves to *Deceased* state. Due to the assumption about the immune mechanism, people in *Recovered* state cannot be infected again, so that they cannot move back to *Susceptible* state.

More precisely, suppose that $t_0$ is the initial time that epidemic was recognized, given a specific day *t* where $t> t_0 > 0$, we denote the functions *S*(*t*), *I*(*t*), *R*(*t*), *D*(*t*) as the numbers of susceptible, infectious, recovered and deceased cases at day *t*, respectively. Moreover, we assume that there are three rate parameters $\beta$, $\gamma$ and $\mu$ of the model as follows.

- $\beta$: the *rate of transmission*, i.e. the average number of contacts of the persons in the community per day (from the first day to the estimated last day of the pandemic).
- $\gamma$: the *rate of recovery*, i.e. the average number of recovered cases in the community per day.
- $\mu$: the *rate of mortality from the epidemic*, i.e. the average number of deceased cases suffering from the infectious cases per day.

Then, given a population of *N* individuals, the SIRD model consists of four ordinary differential equations describing the relationships between the above functions and factors, given in Eqs. (3a)–(3d).

3a $$\begin{aligned} \dfrac{dS}{dt}= &\, {} -\dfrac{{\beta }SI}{N}\end{aligned}$$

3b $$\begin{aligned} \dfrac{dI}{dt}= &\, {} \dfrac{{\beta }SI}{N} - {\gamma }I - {\mu }I\end{aligned}$$

3c $$\begin{aligned} \dfrac{dR}{dt}= &\, {} {\gamma }I\end{aligned}$$

3d $$\begin{aligned} \dfrac{dD}{dt}= &\, {} {\mu }I \end{aligned}$$

The three parameters $\beta$, $\gamma$ and $\mu$, therefore, are very essential to the model. At the early stage, $\beta$ reflects how the infection would increase if individuals were behaving as usual before being informed of medical conditions or any information related to the infection^38^. Moreover, $\beta$ varies depending on how strong the social distancing and hygienic practices that different locations adopt, either because of policy or simply because of voluntary changes in individual behavior^38–41^. Whereas, $\gamma$ provides insights into how many people recover from the epidemic in a period of time. Therefore, the average number of days a person is infected is $\frac{1}{\gamma }$ and we can statistically approximate $\gamma$ based on the average cure time of one person. Finally, $\mu$ represents the average mortality rate in a period of time^38^. Both $\gamma$ and $\mu$ perform the average medical capacity of the region considered.

A unique solution *S*(*t*), *I*(*t*), *R*(*t*), *D*(*t*) for the SIRD model, respect to a certain ($\beta$, $\gamma$, $\mu$), infectious, recovered and deceased cases at the time *t*^38–40^. According to the meaning of ($\beta$, $\gamma$, $\mu$), it is hard to accurately estimate them^42^. In other words, if we can estimate precisely the value of ($\beta$, $\gamma$, $\mu$) from the historical data, we can forecast the trend of the pandemic more exactly. Moreover, those parameters can give us more insightful information about the internal status of the pandemic. For instance, if the number of deceased cases *D*(*t*) still tends to increase in the next days, alongside the high value of $\mu$ while the value of $\beta$ becomes relatively small, one can conclude that the reason for high mortality rate is due to the unbearably serious health status of the infected people. Meanwhile, the transmission in the community now is well-controlled, which allows the authorities to endorse suitable policy (such as lifting the lock-down restriction on the community, if currently applied). Thus, the SIRD model is regarded as an explainable model, which is very helpful for human experts to deal with real situations.

Nonetheless, having an exact ($\beta$, $\gamma$, $\mu$) is not trivial since it depends on many factors such as locations, social policies, region economy, medical capacity, etc. Furthermore, according to the epidemiologists, the parameters can only be approximated by the actual circumference at the location that we consider, since they do not remain unchanged in time.

Thus, when historical data become extremely huge like the real COVID-19 data of the world, it is virtually impossible for human experts to evaluate the value of ($\beta$, $\gamma$, $\mu$) and especially their changes of values when substantial new impacts occur with the recent data. This urges us to consider using Deep Learning approaches to automatically learn and adjust the values of ($\beta$, $\gamma$, $\mu$) from real streaming historical data, resulting in an *Explainable AI model* as subsequently discussed.

## The BeCaked model

**Figure 7:**
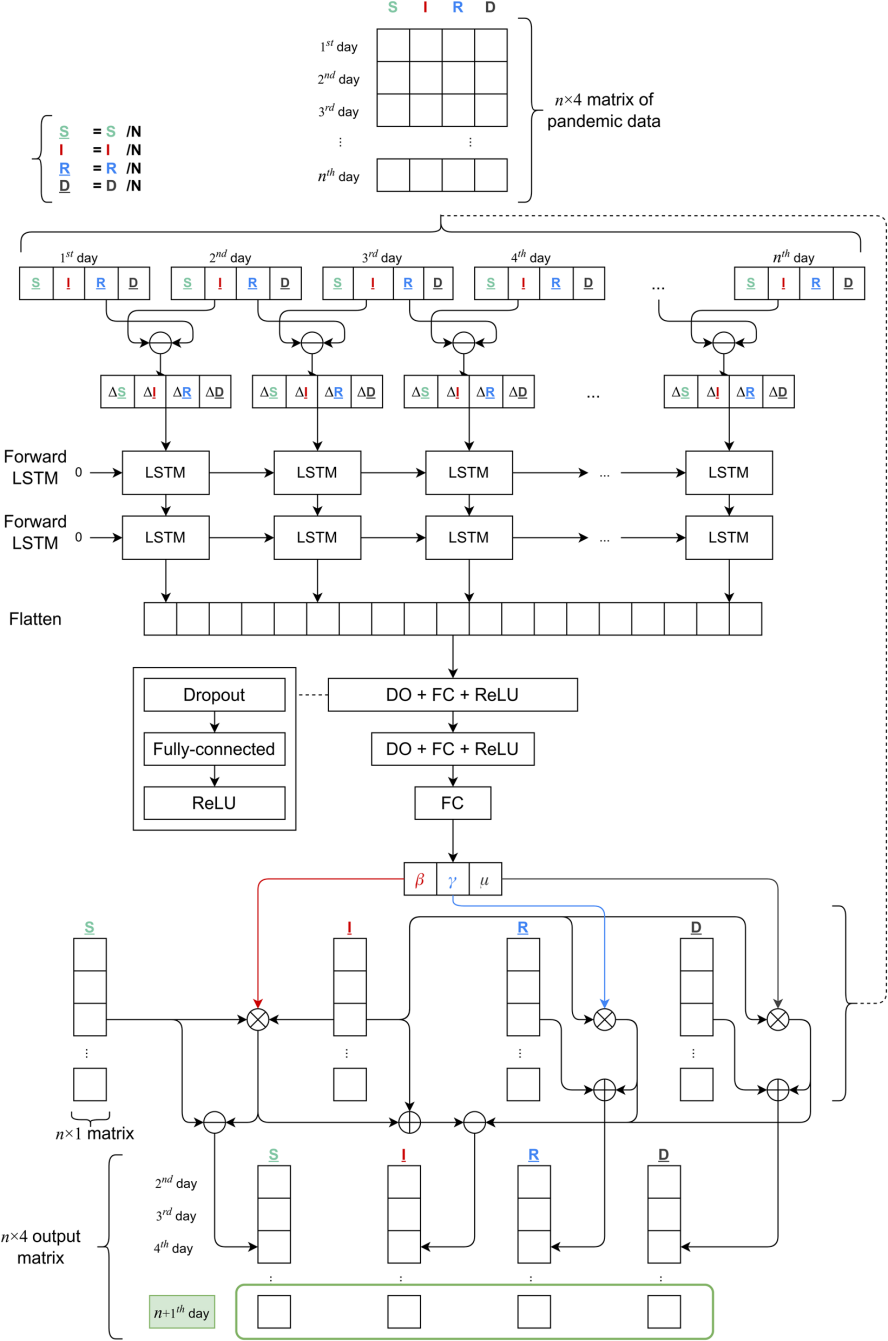
The architecture of BeCaked model.

As mentioned before, the SIRD model can bestow a reasonable explanation regarding internal factors of a pandemic. However, it is very hard to determine the suitable values of the crucial parameters ($\beta$, $\gamma$, $\mu$) of this model from extremely huge sources of historical data. Thus, we enhance the SIRD model by combining it with a Deep Learning architecture to automatically learn the suitable values of those parameters. As a result, we obtain a hybrid model, known as *BeCaked*, as presented in Fig. 7. The general ideas of employing Deep Learning techniques in BeCaked are as follows.

- We firstly use LSTM to make predictions of future values by extracting significant features from the input of historical sequential data.
- We combine LSTM with VAE to encode the predicted output as the desired parameters of ($\beta$, $\gamma$, $\mu$) and use the backpropagation capability of the end-to-end neural network to train the suitable values of those parameters from the input data. We also take advantage of the semi-supervised learning mechanism of VAE to auto-label the training data, as discussed later.

Particularly, in order to deal with the huge volumes of data, our main goal is to also reduce computation costs, simplify the loss function and explain the forecasting results. We carry out this goal in our proposed model architecture shown in Fig. 7. The detailed descriptions of BeCaked are discussed as follows.

### Input data

Input data of the BeCaked model is a $n\times 4$ matrix, where *n* indicates the last recent *n* days to be studied, assumed from 1st to *n*th day. The *i*th row of this matrix is a 4-dimension vector of (*S*(*i*), *I*(*i*), *R*(*i*), *D*(*i*)) of the corresponding *i*th day, whose meanings had been already explained previously.

### Feature extraction for LSTM layers

From the raw information given from the historical input data and the population *N*, we then produce feature vectors $V_i=(\Delta {\underline{S}}_i, \Delta {\underline{I}}_i, \Delta {\underline{R}}_i, \Delta {\underline{D}}_i)$, where $\Delta {\underline{S}}_i$, $\Delta {\underline{I}}_i$, $\Delta {\underline{R}}_i$, and $\Delta {\underline{D}}_i$ can be observed in Eqs. (4a)–(4d).

4a $$\begin{aligned} \Delta {\underline{S}}_i= &\, {} \dfrac{S(i+1)}{N} - \dfrac{S(i)}{N}\end{aligned}$$

4b $$\begin{aligned} \Delta {\underline{I}}_i= &\, {} \dfrac{I(i+1)}{N} - \dfrac{I(i)}{N}\end{aligned}$$

4c $$\begin{aligned} \Delta {\underline{R}}_i= &\, {} \dfrac{R(i+1)}{N} - \dfrac{R(i)}{N}\end{aligned}$$

4d $$\begin{aligned} \Delta {\underline{D}}_i= &\, {} \dfrac{D(i+1)}{N} - \dfrac{D(i)}{N} \end{aligned}$$

Finally, the sequence of feature vector $\{V_i\}$ will be used as the input for the LSTM layers. In BeCaked, we use two stacked layers of LSTM to increase the abstraction capability from the extracted features.

### Parameter encoding

The output of LSTM layers are then flattened as a 1-dimension vector, from which we encode into ($\beta$, $\gamma$, $\mu$) parameters. The encoding process is carried out by the typical MLP technique, including two FC layers enhanced with drop-out techniques and ReLU activation functions employed.

Thus, the BeCaked model can be regarded as a variation of the VAE model whose encoder consists of LSTM layers and FC layers previously described. The output of this encoder is then the parameters of ($\beta$, $\gamma$, $\mu$), trainable by the model decoder as subsequently discussed.

### Decoding process

From the encoded parameters of ($\beta$, $\gamma$, $\mu$), the decoder will attempt to produce the desired output, which is also a $n\times 4$ matrix similar to the input matrix. However, the output matrix captures the information from 2nd to $(n+1)$th day from the historical data. Thus, the labeling process for our encoder-decoder mechanism can be done automatically, like all other VAE systems.

In order to decode the output matrix from the three parameters ($\beta$, $\gamma$, $\mu$) learned with the *n*-day input data, the decoder approximates the SIRD model with the *Euler* method^43^ because it is the easiest but most efficient way for approximating differential equations. We present the equations for approximating the SIRD model using the Euler method with step $h=1$ in Eqs. (5a)–(5d). The reason why we choose Euler instead of *Runge-Kutta*^44^ or other methods is that it is the most suitable solving method for the data we have and step $h=1$ is corresponding to a day in the data.

5a $$\begin{aligned} \dfrac{S(i+1)}{N}= &\, {} \dfrac{S(i)}{N} - \beta \dfrac{S(i)}{N} \dfrac{I(i)}{N}\end{aligned}$$

5b $$\begin{aligned} \dfrac{I(i+1)}{N}= &\, {} \dfrac{I(i)}{N} + \beta \dfrac{S(i)}{N} \dfrac{I(i)}{N} - \gamma \dfrac{I(i)}{N} - \mu \dfrac{I(i)}{N}\end{aligned}$$

5c $$\begin{aligned} \dfrac{R(i+1)}{N}= &\, {} \dfrac{R(i)}{N} + \gamma \dfrac{I(i)}{N}\end{aligned}$$

5d $$\begin{aligned} \dfrac{D(i+1)}{N}= &\, {} \dfrac{D(i)}{N} + \mu \dfrac{I(i)}{N} \end{aligned}$$

In Eqs. (5a)–(5d), $S(i+1)$, $I(i+1)$, $R(i+1)$, $D(i+1)$ represent the number of susceptible, infectious, recovered, and deceased cases at the $(i+1)$th day which is right after the *i*th day, respectively.

In order to simplify the model, we normalize all data by dividing them for the population *N*. Consider we have normalized functions as in Eqs. (6a)–(6d).

6a $$\begin{aligned} {\underline{S}}(i)= &\, {} \dfrac{S(i)}{N}\end{aligned}$$

6b $$\begin{aligned} {\underline{I}}(i)= &\, {} \dfrac{I(i)}{N}\end{aligned}$$

6c $$\begin{aligned} {\underline{R}}(i)= &\, {} \dfrac{R(i)}{N}\end{aligned}$$

6d $$\begin{aligned} {\underline{D}}(i)= &\, {} \dfrac{D(i)}{N} \end{aligned}$$

Then Eqs. (5a)–(5d) can be written as Eqs. (7a)–(7d).

7a $$\begin{aligned} {\underline{S}}(i+1)= &\, {} {\underline{S}}(i) - \beta {\underline{S}}(i) {\underline{I}}(i)\end{aligned}$$

7b $$\begin{aligned} {\underline{I}}(i+1)= &\, {} {\underline{I}}(i) + \beta {\underline{S}}(i) {\underline{I}}(i) - \gamma {\underline{I}}(i) - \mu {\underline{I}}(i)\end{aligned}$$

7c $$\begin{aligned} {\underline{R}}(i+1)= &\, {} {\underline{R}}(i) + \gamma {\underline{I}}(i)\end{aligned}$$

7d $$\begin{aligned} {\underline{D}}(i+1)= &\, {} {\underline{D}}(i) + \mu {\underline{I}}(i) \end{aligned}$$

### Training process

In the training process, after the input data goes through all layers of our model, we calculate the *Mean Squared Error* (MSE)^45^ loss function (Eq. (8)) between the output of BeCaked and the real data. The reason that we choose MSE is that it is simple and reflects the true error rate between the real data and the forecasting results, which is better for optimization purpose, compared to *Root Mean Squared Error* (RMSE) or other loss functions. Then we use *Adam optimizer*^36^ to update our model weights because it is the most suitable for noisy data. Let $Y_i$ and ${\widehat{Y}}_i$ be the *i*th vectors from actual data and the predicted values of BeCaked, w.r.t the $n\times 4$ output matrix discussed in the decoding process, the MSE of an epoch in the training process is evaluated as (8).

8 $$\begin{aligned} MSE = \frac{1}{n}\sum _{i=1}^{n} (Y_i - {\hat{Y}}_i)^2 \end{aligned}$$

Since BeCaked is an end-to-end VAE neural network as previously described, the loss function given in Eq. (8) can be used to update the weights of the whole system by the typical backpropagation manner (note that the decoder does not use any trainable weights and will not be updated during the training process). When the loss values become stable, the training process is converged and BeCaked is now able to predict ($\beta$, $\gamma$, $\mu$) from any given sources of real input data.

### Explainability of BeCaked

As previously described, the basic operational mechanism of BeCaked is taking ($\beta$, $\gamma$, $\mu$) and data in the previous days to calculate the number of susceptible, infectious, recovered, and deceased cases in the next days. The most important thing that helps this model be successful is choosing the correct features of the input data so that the model can learn how to estimate ($\beta$, $\gamma$, $\mu$) in the best way. In other words, BeCaked can serve not only as a regression system with the predicted values for the future, but also as a VAE generating the parameters of ($\beta$, $\gamma$, $\mu$) with an explanation of the regressed value at the same time.

Also, the key idea which makes our model explainable is the way we infer the value of ($\beta$, $\gamma$, $\mu$). On the one hand, our decoder ensures that the inferred values of ($\beta$, $\gamma$, $\mu$) are mathematically correct to give accurate predictions on the training data. On the other hand, BeCaked manipulates the encoding process by taking into account the relationship between the difference in the number of considered cases in the previous days and the ($\beta$, $\gamma$, $\mu$) of the following days. This is a varied flow correlation^39–41^, so if the more quickly the difference in the number of cases in the previous day increases, the higher the corresponding parameter is. For example, if we have recovered cases in three consecutive days as respective (5, 10, 20) the recovery rate will be considered increasing. Meanwhile, if the recovered cases are (5, 10, 12), the recovery case can be regarded as decreasing even though the number of recovered people still keeps increasing daily.

## Performance evaluation

### Data preparation and pre-processing

In this evaluation process, we used the dataset provided by the *Johns Hopkins University Center for System Science and Engineering* (JHU CSSE)^46,47^. This dataset is collected from January 2020 until now from various sources such as the *World Health Organization* (WHO), *European Centre for Disease Prevention and Control* (ECDC), *United States Centre for Disease Prevention and Control* (US CDC), etc.^46,47^. In detail, this dataset contains the daily number of total infectious (including recovered and deceased cases), recovered, and deceased cases in all countries around the world.

In our experiment, we used the data from January 2020 to the end of June 2020 as training data and the July 2020 data for the testing period. As presented in “The BeCaked model”, we need the input containing four values in each day: susceptible, infectious, recovered, and deceased, but with the above dataset, we only have total infectious, recovered, and deceased cases. Therefore, we need to have a total population of the world and recalculate all the required input. The data about world population is provided by Worldometers^48^. Equations (9a)–(9d) show how to calculate the input for BeCaked model from the dataset.

9a $$\begin{aligned} Input_{Susceptible}= &\, {} Total\_Population - Total\_Infectious\end{aligned}$$

9b $$\begin{aligned} Input_{Infectious}= &\, {} Total\_Infectious - Recovered - Deceased\end{aligned}$$

9c $$\begin{aligned} Input_{Recovered}= &\, {} Recovered\end{aligned}$$

9d $$\begin{aligned} Input_{Deceased}= &\, {} Deceased \end{aligned}$$

The input of our model are then normalized into percentages by dividing all data for the total population to match with the input of our proposed method described in “The BeCaked model”.

### Global evaluation

In the evaluation process, to choose the most optimal day lag number *n*, we conduct the experiments on global using different day lag numbers. According to the result of McAloon’s study (2020)^49^, the day lag number varies from 5 to 14 days, so that, we test our model with 7, 10 and 14 day lag to find the most suitable one.

To determine the suitable value *n* of the lag days, we used the *recursive stategy*^50^ to perform *k*-step forecasting. In details, a *k*-step forecasting process is described as follows. Firstly, we only use *n*-day data (June $(30-n+1)$th–June 30th) as the initial input for forecasting the next $n+1$th day (the number *n* is corresponding to *n*-day lag), which *n* is set as 7, 10, 14, respectively. Then, we repeat that process *k* times. At each step, we predict the number of cases (susceptible, infectious, recovered, deceased) for the next day and use it (our predicted cases) as the input for the next iteration. In this process, because the testing data is July 2020 data, the number step *k* varies from 1 to 31, we eventually choose *k* as the possible maximal value of 31.

In Table 1, we have shown our forecast results using 7, 10 and 14 day lag in *R Squared* ($R^2$) (Eq. (10)) and *Mean Absolute Percentage Error* (*MAPE*) (Eq. (11)) metric.

10 $$\begin{aligned} R^2 = 1 - \frac{RSS}{TSS} \end{aligned}$$

The *RSS*, *TSS* in Eq. (10) denote for the *Residual Sum of Squares* and the *Total Sum of Squares*. The $R^2$ metric given in Eq. (10) provides an insight into the similarity between real and predicted data. The closer to 1 the $R^2$ is, the more explainable the model is. The *MAPE* given in Eq. (11) tells us about the mean of the total percentage errors for *k*-step forecasting. If the value of this *MAPE* metric is closer to 0, it indicates the better results.

11 $$\begin{aligned} MAPE = \frac{1}{k}\sum _{i=1}^{k} \frac{|Y_i - {\hat{Y}}_i|}{Y_i} \end{aligned}$$

In Eq. (11), *k*, $Y_i$, ${\hat{Y}}_i$ denote for the number of steps, the actual cases and our predicted cases, respectively.

**Table 1:** Comparison of BeCaked performance with 7, 10 and 14 day lag.

	7-day	10-day	14-day
**Total infectious cases**			
$R^2$	0.77284	$\mathbf {0.99790}$	0.95795
*MAPE*	0.05592	$\mathbf {0.00509}$	0.02552
**Daily infectious cases**			
$R^2$	0.89739	$\mathbf {0.98050}$	0.87828
*MAPE*	0.12455	$\mathbf {0.01268}$	0.05842
**Recovered cases**			
$R^2$	0.45429	$\mathbf {0.98784}$	0.67187
*MAPE*	0.10912	$\mathbf {0.01869}$	0.08676
**Deceased cases**			
$R^2$	$-22.25663$	$\mathbf {0.98904}$	$-12.13873$
*MAPE*	0.32878	$\mathbf {0.00676}$	0.24510

Best results are in [bold].

**Figure 8:**
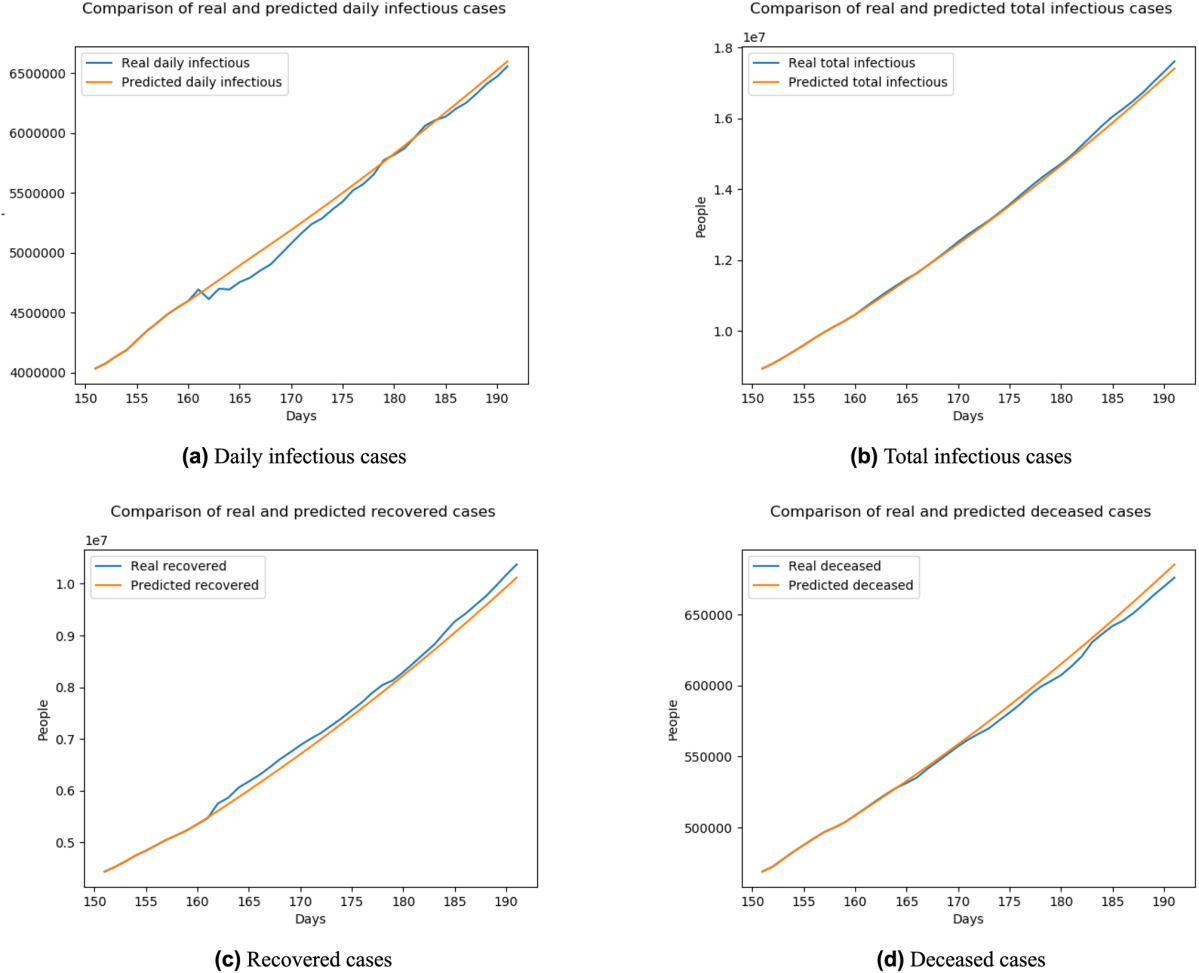
Comparison of the number of cases between real data and BeCaked forecasting results from 161st–191st day (Jul. 1st 2020–Jul. 31st 2020) of the world.

**Figure 9:**
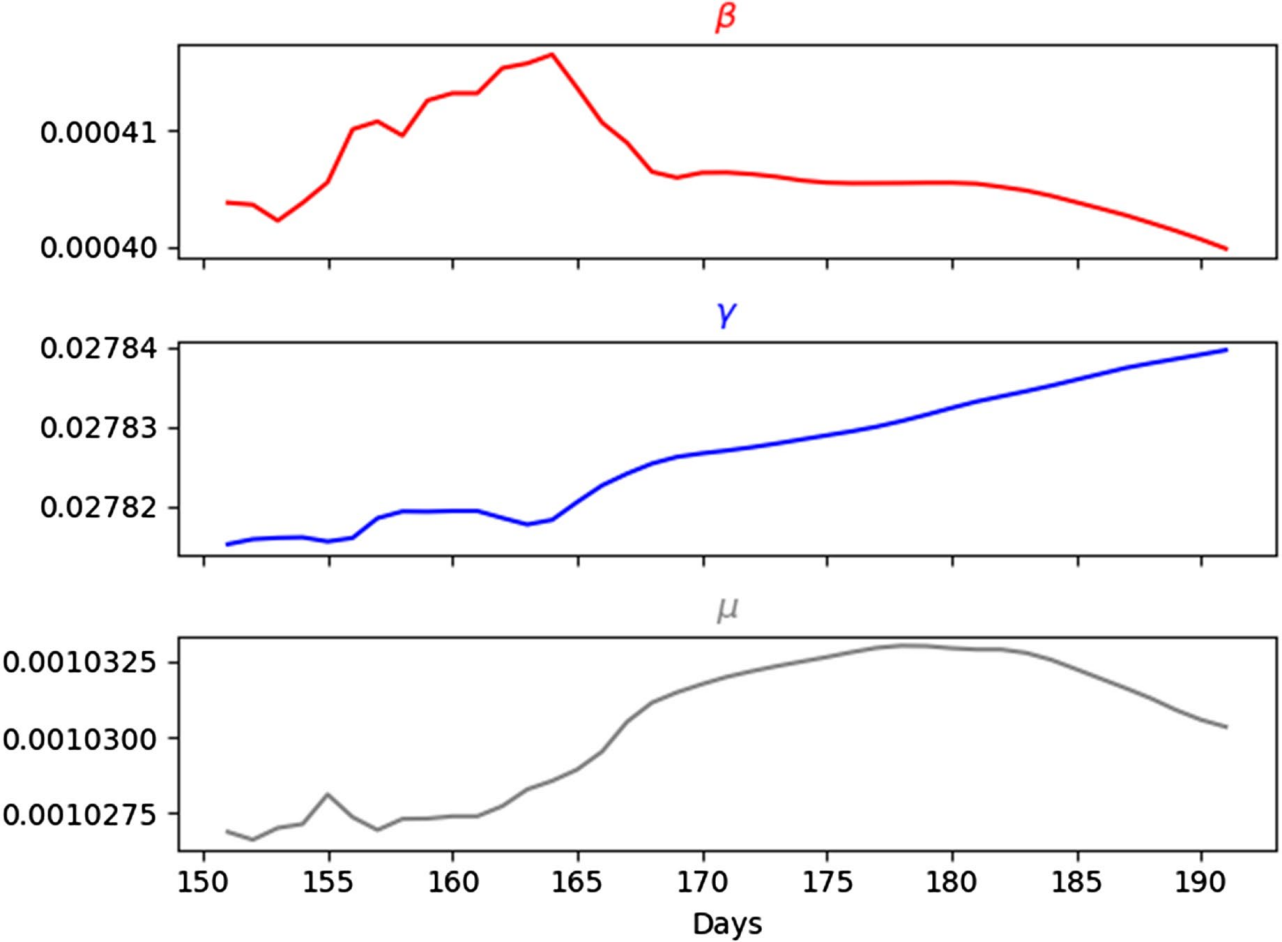
Predicted transition rates from 161st–191st day (Jul. 1st 2020–Jul. 31st 2020) of the world.

According to the results shown in Table 1, the highest performance of our model was achieved with 10-day lag, so that we chose the number of day lag as 10 for further evaluations. We also visualized our results for a better overview using 10-day lag. Figure 8a shows the comparison of daily infectious cases between real data and BeCaked forecasting results while Fig. 8b shows that of the total infectious cases. Also, the comparison of recovered and deceased cases are presented in Fig. 8c,d, respectively. Moreover, we visualized the ($\beta$, $\gamma$, $\mu$) corresponding to the results of forecasting in Fig. 9. In this period (July 01st–July 31st), this pandemic in many countries started to be controlled, so that the overall transmission rate decreased. Due to the slower speed of transmission, the recovery rate increased. The hidden truth here is that the health system in those countries was load-reduced and doctors could pay more attention to currently infected patients. Because of the above reasons, the mortality rate decreased too.

In Table 3, we compared our model with some top-tier forecasting-specialized others from statistical models to machine learning models including *Autoregressive Integrated Moving Average* (ARIMA)^12^, *Ridge*^51^, *Least Absolute Selection Shrinkage Operator* (LASSO)^52^, *Support Vector Machine for Regression* (SVR)^53^, *Decision Tree Regression* (DTR)^54^, *Random Forest Regression* (RFR)^55^ and *Gradient Boost Regression* (GBR)^56^. Except for ARIMA, the other models use the same number of day lag *n* as our BeCaked does (which is 10) to forecast the future. The specific configurations of each model are listed in Table 2. Even though these models are widely used in predicting the future for time-series data and achieving comparative results^50^, in the case of the COVID-19 long time forecasting problem, with the exception of our model, only Ridge and LASSO have acceptable results. Our model is proved to attain overall comparative performance with those mentioned methods by these results given below.

**Table 2:** Configurations of top-tier forecasting-specialized methods.

Model	Configurations
ARIMA	$p=1; d=0; q=0;$
Ridge	$\alpha =1; solver=svd; tolerance=10^{-3};$
LASSO	$\alpha =1; max\_iter=1000; selection=cyclic; tolerance=10^{-4};$
SVR	$kernel=rbf; \gamma =scale; C=1; \epsilon =0.1; tolerance=10^{-3};$
DTR	$criterion=mse; splitter=best; min\_samples\_split=2; min\_samples\_leaf=1;$
RFR	$n\_estimators=100; criterion=mse; min\_samples\_split=2; min\_samples\_leaf=1;$
GBR	$loss=least\_squared; n\_estimators=100; criterion=friedman\_mse;$
	$$min\_samples\_split=2; min\_samples\_leaf=1; max\_depth=3; tolerance=10^{-4};$$

**Table 3:** Comparison of BeCaked and top-tier forecasting-specialized methods with 31-step forecast.

	BeCaked	ARIMA	Ridge	LASSO	SVR	DTR	RFR	GBR
**Total infectious cases**								
$R^2$	0.99790	$-2.84161$	0.98777	$\mathbf {0.99907}$	$-18.11617$	$-2.84160$	$-2.99678$	$-2.84598$
*MAPE*	0.00509	0.23243	0.01346	$\mathbf {0.00112}$	0.62084	0.23243	0.23929	0.23262
**Daily infectious cases**								
$R^2$	$\mathbf {0.98050}$	$-2.31481$	0.39289	0.92269	$-29.01740$	$-2.31481$	$-2.44266$	$-2.32188$
*MAPE*	0.01268	0.38969	0.03052	$\mathbf {0.00350}$	0.78887	0.38969	0.39887	0.38998
**Recovered cases**								
$R^2$	$\mathbf {0.98784}$	$-3.07765$	0.76234	0.96656	$-13.46395$	$-3.07765$	$-3.24544$	$-3.08040$
*MAPE*	0.01869	0.29221	0.07195	$\mathbf {0.01153}$	0.64754	0.29221	0.30097	0.29236
**Deceased cases**								
$R^2$	$\mathbf {0.98904}$	$-2.81195$	0.88473	0.53645	$-46.32486$	$-2.81195$	$-2.95304$	$-2.82239$
*MAPE*	$\mathbf{0}.00676$	0.13248	0.01988	0.04018	0.55858	0.13248	0.13594	0.13274

Best results are in [bold].

### Country evaluation

At the country level, we also conducted the same evaluation process as we did in global scale. We chose six countries with different locations, social policies, and anti-epidemic strategies, etc. for testing our model in various conditions.

Firstly, we fit our model for each country until the end of June 2020. Then, we used the July 2020 cases for the testing phase. In this comparison, we used the same methods with configurations as we did at the whole world level. These below evaluations were done in 1-step (Table 4), 7-step (Table 5) and 15-step (Table 6) forecasting using the daily infectious cases. The reason why we did more comparisons will be discussed in “Discussion”.

In July 2020, many countries began to have better control of the development of this COVID-19 pandemic by restricting outside agents from spreading viruses. However, some countries have reopened after lockdown, such as the United State, Australia, Italy, etc. This became a favorable condition for external factors to directly influence the increase in the number of cases in those countries. Therefore, to effectively forecast the long-time situation of those countries, a forecasting model must have the ability to adapt to emerging changes in the pandemic exponential growth rate. Due to that, the compared results below reflected the strong adaptive capacity of our BeCaked model along with others.

**Table 4:** Comparison of BeCaked and top-tier forecasting-specialized methods at country level with 1-step forecast.

	BeCaked	ARIMA	Ridge	LASSO	SVR	DTR	RFR	GBR
**Australia**								
$R^2$	0.99112	0.98329	0.99337	$\mathbf {0.99531}$	$-0.48798$	0.98342	0.96787	0.98299
*MAPE*	0.04703	0.10236	$\mathbf {0.01093}$	0, 04078	0.85609	0.10204	0.14942	0.10344
**Italy**								
$R^2$	$\mathbf {0.96381}$	0.94660	0.92646	0.88220	$-22.56497$	0.94889	0.90082	0.94876
*MAPE*	0.00496	0.01655	$\mathbf {0.00024}$	0.05999	0.93214	0.01684	0.02570	0.02165
**Russia**								
$R^2$	0.92922	0.91663	$\mathbf {0.98479}$	0.92367	$-108.89720$	0.91643	0.87353	0.91854
*MAPE*	$\mathbf {0.00004}$	0.02856	0, 00007	0.00938	0.77448	0.02851	0.04442	0.02938
**Spain**								
$R^2$	$\mathbf {0.98765}$	0.97214	0.98591	0.98362	$-10.84390$	0.97212	0.94968	0.97053
*MAPE*	$\mathbf {0.00174}$	0.01418	0.00325	0.01881	0.74045	0.01406	0.02173	0.01522
**United Kingdom**								
$R^2$	$\mathbf {0.99833}$	0.98713	0.99764	0.96375	$-449.89408$	0.98748	0.96991	0.98463
*MAPE*	0.00030	0.00255	$\mathbf {0.00014}$	0.00437	0.52586	0.00249	0.00397	0.00282
**United States**								
$R^2$	$\mathbf {0.99688}$	0.98676	0.99648	0.99461	$-9.99942$	0.98676	0.96834	0.98633
*MAPE*	0.00552	0.02644	$\mathbf {0.00004}$	0.00069	0.62264	0.02642	0.04185	0.02687

Best results are in [bold].

**Table 5:** Comparison of BeCaked and top-tier forecasting-specialized methods at country level with 7-step forecast.

	BeCaked	ARIMA	Ridge	LASSO	SVR	DTR	RFR	GBR
**Australia**								
$R^2$	0.88112	0.74039	$\mathbf {0.99115}$	0.98878	$-0.94255$	0.73989	0.68456	0.73797
*MAPE*	0.18610	0.32529	$\mathbf {0.06932}$	0.11617	0.88976	0.32524	0.36291	0.32645
**Italy**								
$R^2$	$\mathbf {0.89690}$	0.66110	$-0.20361$	0.63946	$-20.00836$	0.69335	0.53913	0.69311
*MAPE*	0.02365	0.06104	$\mathbf {0.00444}$	0.12168	0.93036	0.06223	0.06936	0.06644
**Russia**								
$R^2$	0.85845	0.70449	$\mathbf {0.92995}$	0.84984	$-106.05299$	0.70217	0.59478	0.71031
*MAPE*	0.00222	0.10383	$\mathbf {0.00087}$	0.02079	0.77662	0.10370	0.11736	0.10442
**Spain**								
$R^2$	0.98013	0.68411	$\mathbf {0.98210}$	0.93588	$-12.06027$	0.68644	0.61741	0.68019
*MAPE*	$\mathbf {0.00836}$	0.05367	0.01601	0.04576	0.74972	0.05306	0.05929	0.05420
**United Kingdom**								
$R^2$	$\mathbf {0.99413}$	0.77401	0.98469	0.84731	$-456.43229$	0.77985	0.72832	0.76877
*MAPE*	$\mathbf {0.00119}$	0.01027	0.00164	0.00918	0.52910	0.01004	0.01131	0.01037
**United States**								
$R^2$	0.98679	0.79943	$\mathbf {0.98702}$	0.97658	$-11.07041$	0.79943	0.74460	0.79768
*MAPE*	0.01900	0.09879	$\mathbf {0.00080}$	0.00067	0.64423	0.09871	0.11258	0.09912

Best results are in [bold].

**Table 6:** Comparison of BeCaked and top-tier forecasting-specialized methods at country level with 15-step forecast.

	BeCaked	ARIMA	Ridge	LASSO	SVR	DTR	RFR	GBR
**Australia**								
$R^2$	0.09703	$-0.10753$	$\mathbf {0.92366}$	0.86414	$-1.69800$	$-0.12140$	$-0.20682$	$-0.12229$
*MAPE*	0.40278	0.50951	$\mathbf {0.18428}$	0.26379	0.92842	0.51087	0.53734	0.51162
**Italy**								
$R^2$	$\mathbf {0.61706}$	$-0.07923$	$-10.41220$	$-2.08983$	$-16.35794$	0.07287	$-0.17657$	0.07720
*MAPE*	0.05745	0.10709	$\mathbf {0.01292}$	0.23014	0.92750	0.10942	0.11508	0.11293
**Russia**								
$R^2$	$\mathbf {0.27693}$	$-0.09658$	$-0.28502$	$-0.71668$	$-102.60031$	$-0.09129$	$-0.19068$	$-0.07648$
*MAPE*	0.01246	0.18125	$\mathbf {0.00158}$	0.05668	0.77912	0.18115	0.19226	0.18170
**Spain**								
$R^2$	$\mathbf {0.90753}$	$-0.19684$	0.65409	0.38282	$-13.40342$	$-0.22237$	$-0.28920$	$-0.23309$
*MAPE*	$\mathbf {0.01356}$	0.09955	0.06038	0.11265	0.75909	0.09886	0.10272	0.09994
**United Kingdom**								
$R^2$	$\mathbf {0.99278}$	$-0.06679$	0.90663	$-0.10126$	$-465.84698$	$-0.02151$	$-0.12357$	$-0.04241$
*MAPE*	$\mathbf {0.00268}$	0.02080	0.00436	0.02445	0.53362	0.02024	0.02146	0.02054
**United States**								
$R^2$	0.94760	$-0.04783$	$\mathbf {0.96380}$	0.93578	$-12.74072$	$-0.04770$	$-0.15771$	$-0.05102$
*MAPE*	0.04370	0.18476	0.00917	$\mathbf {0.00477}$	0.67073	0.18467	0.19654	0.18503

Best results are in [bold].

**Figure 10:**
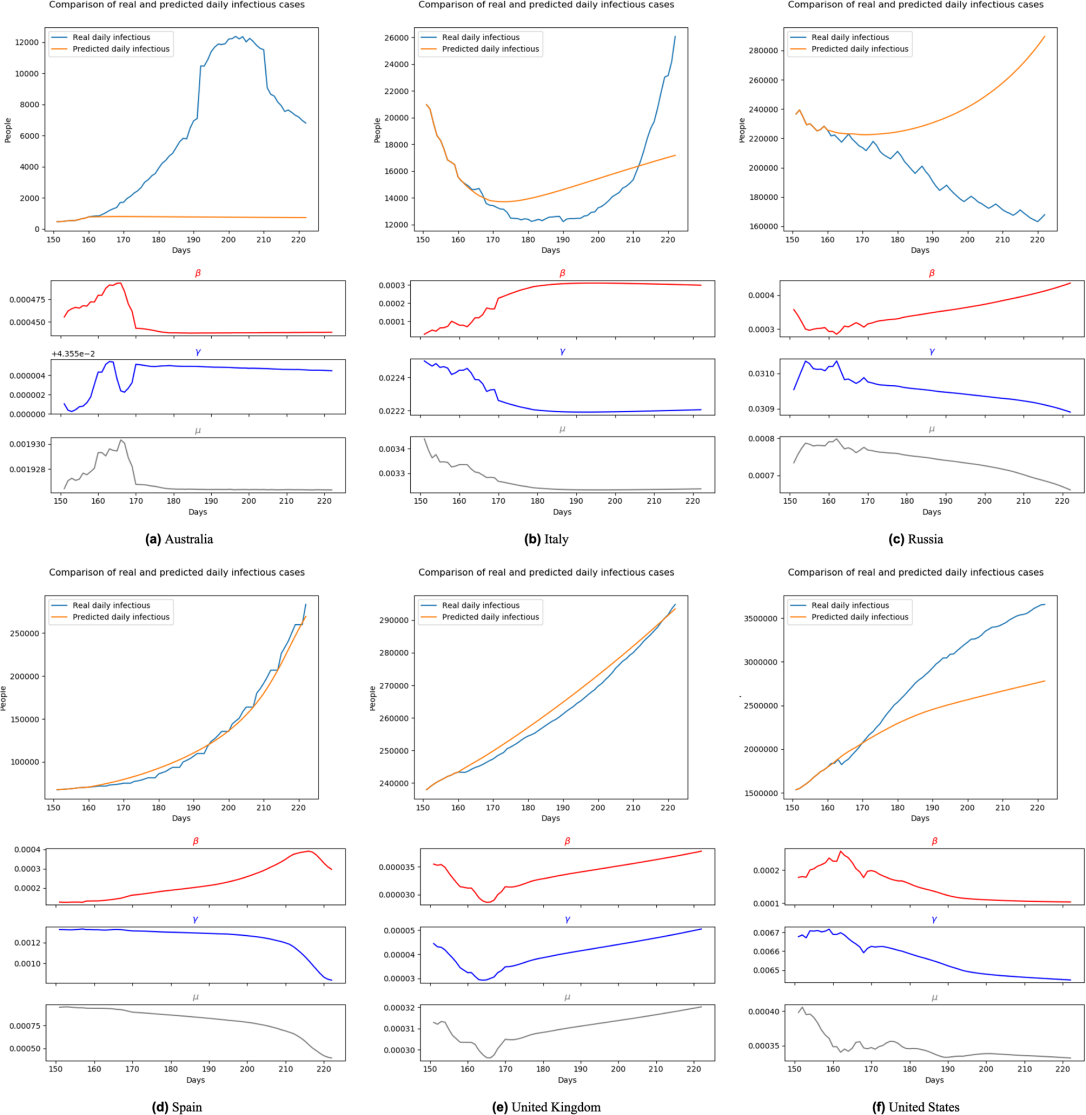
Forecasting results with transition rates of 161th–222nd day (Jul. 1st 2020–Aug. 31st 2020) using data of 151st–160th day (Jun. 21st 2020–Jun. 30th 2020).

**Figure 11:**
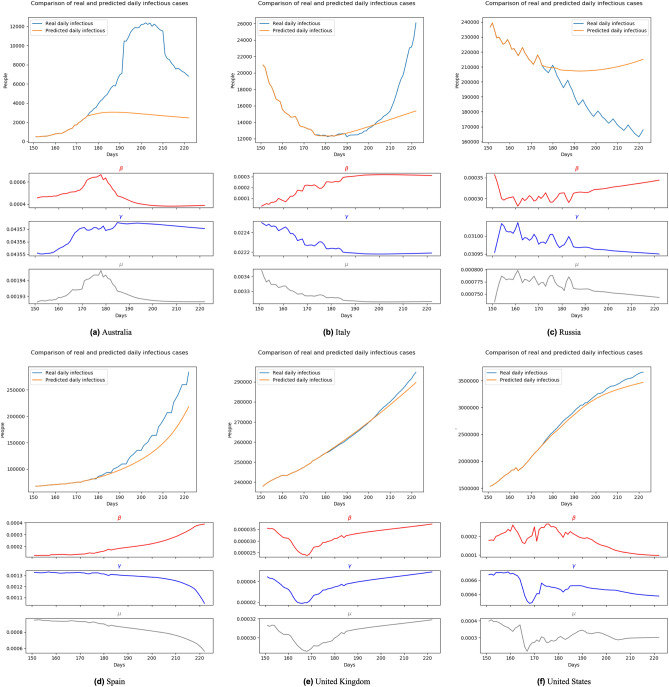
Forecasting results with transition rates of 176th–222nd day (Jul. 16th 2020–Aug. 31st 2020) using data of 166th–175th day (Jul. 6th 2020–Jul. 15th 2020).

**Figure 12:**
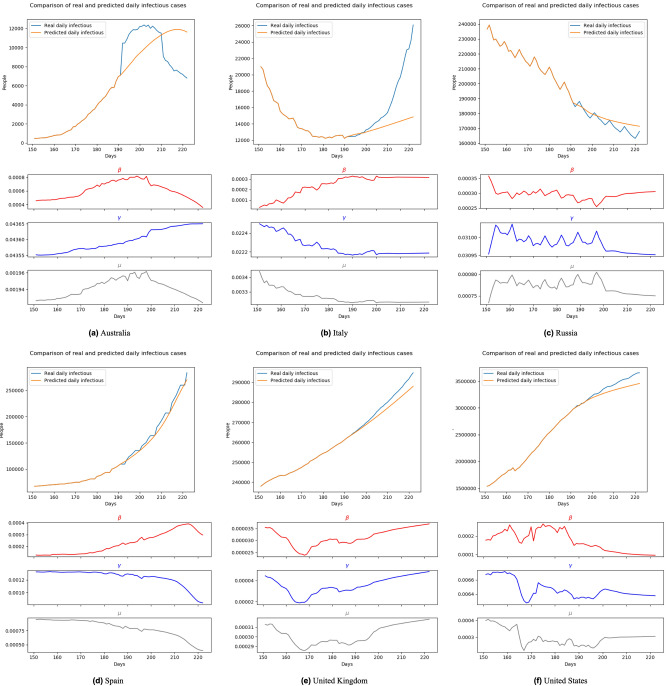
Forecasting results with transition rates of 192th–222nd day (Aug. 1st 2020–Aug. 31st 2020) using data of 182nd–191th day (Jul. 22nd 2020–Jul. 31st 2020).

**Figure 13:**
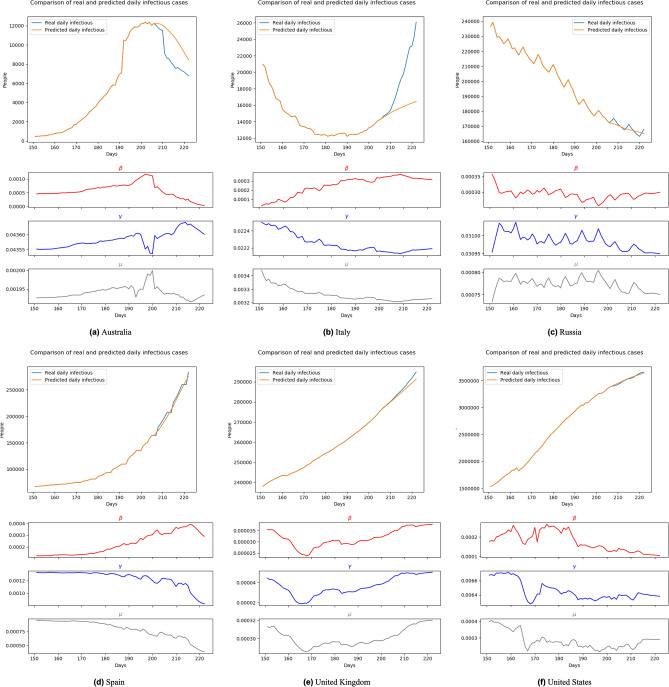
Forecasting results with transition rates of 207th–222nd day (Aug. 16th 2020–Aug. 31st 2020) using data of 197th–206th day (Aug. 6th 2020–Aug. 15th 2020).

For a more challenging evaluation, we kept the training set to the end of June 2020 while stimulating the progress of long-term forecasting. In detail, firstly, we used 10 final days of June 2020 cases as the initial input and predict the pandemic in July and August 2020. On the next day in stimulating, we used the data of nine last days of June and July 1st as the input and re-produce the forecasting until the end of August. This process is the same as the natural behavior of most real-life forecast systems as we need to re-run the forecasting each day to get the most accurate result. With this testing, our model shows not only its performance but also its capacity of catching the changes of the pandemic. Figures 10, 11, 12 and 13 show the predicting results of daily infectious cases at the beginning and middle of July and August 2020, respectively. According to these figures, we can observe that, like other prediction models, BeCaked only works well when given sufficient input of historical data. As a result, in Fig. 13, BeCaked enjoys good accuracy in all countries when forecasting.

Moreover, unlike other blackbox-like prediction models, BeCaked can also provide an explanation for its results, in terms of the parameters ($\beta$, $\gamma$, $\mu$). For example, in Fig. 13, let us consider the cases of Spain and the United Kingdom. Even though the predicted curves of those two countries run in similar shapes, their parameters tell us different stories. The common things between those two countries are that they failed to control the transmission rate (maybe their lock-down policy did not make sufficient impacts). However, in Spain, they had been suffering from a high mortality rate for a long time, showing that their health system had difficulty in dealing with a large number of infectious cases, even though the recovery rate had also been increasing (i.e. more patients were cured daily). In contrast, in the United Kingdom, the rates of recovery cases and mortality cases gradually reduced at the early stage, indicating that the government somehow well controlled the situation in this period, which was also implied by the reduction of the transmission rate. However, when the transmission rate began to increase (corresponding to the time the lock-down policy had been relaxed in this country), the situation had been worse quickly in terms of mortality. At the end of this experiment, even though the regression models of two countries generate two similar shapes, like previously discussed, the parameters ($\beta$, $\gamma$, $\mu$) indicate that Spain already controlled the situation and things would be improved. Meanwhile, the United Kingdom still had a hard time awaiting ahead. Real data taken afterward confirmed our predictions.

In other countries, BeCaked can also be able to tell us what happened “behind the scenes” of the generated results. In Australia, the major turn occurred when the government succeeded in controlling the transmission rate by their lock-down policy. From that point, the recovery rate increased and the mortality rate decreased in this country, leading to the stable situation they are enjoying now.

Meanwhile, in Russia, things are up and down many times, reflecting the rapid policy changing of this country during this period. However, this country generally attempts to maintain less direct contact in the community to reduce the transmission rate, which makes our model promise a better situation for them.

In the United States, their social distancing policies had been somehow proved effective when both transmission rate and mortality rate had gradually reduced. However, the absolute number of infectious cases has still stably increased, which can be explained by the reducing number of recovery cases. This shows that the country was struggling to handle the infected patients in the previous days of the outbreak.

In a broader view, we can consider that Spain and the United Kingdom had an almost unchanged policy which leads to a familiar situation, so that our model can predict their pandemic future accurately in the very early time. The evidence for this is that the shape of $\beta$, $\gamma$, $\mu$ line of these two nations at Fig. 10, 11, 12 and 13 are almost the same. Australia and the United States, in the past, faced the same situation but they did not provide any actions or policies to prevent external factors from spreading the virus. This is the reason why their infectious case increased dramatically. But, because in the past, they have faced this situation, our model can give good forecast results after “realizing” this situation (after about 30 days). Considering the parameter lines of the two above nations, we can see that in the stimulating progression, they are not stable. But in general, their directions are the same as the first forecast. Towards Italy and Russia, our model takes a little bit more time to change the direction of the forecast line, due to the strange situation. We can get this by comparing the parameter lines in Figs. 10, 11, 12 and 13 of Australia and Russia. The direction of these lines has changed as the policies of these two nations become loose. To be simple, it is because there is no pattern of this situation in the training data (pandemic data until the end of June 2020).

With the above result, we can consider that our proposed solution can catch up with the change in the pandemic situation. With the unchanged training set, our model can give very good forecast results if the situation is more stable. In case a strange situation occurs, our model needs time to give a more accurate forecast.

In the real-life application, the forecasting models are updated regularly using reinforcement learning methods in order to make them more “update” to new situations. Therefore, to get better results of our model in real-life, we need to finetune it with new data every one or two weeks.

## Discussion

### Forecasting results

Forecasting a pandemic has never been easy, especially for this COVID-19 situation. The effectiveness of a forecasting model not only comes from the exact results but also the explanation or the root cause of those predicted numbers. Until now, almost no pure mathematical or machine learning model can achieve that double standard. Therefore, we tried to combine both models to create an Explainable AI one to solve that problem. The combination of Variational Autoencoder and SIRD we have constructed can overcome the limitations of each other and take advantage of semi-supervised learning to be more efficient in the training process.

With the support of Deep Learning, based on the number of infectious, recovered and deceased cases, our BeCaked model can determine the ($\beta$, $\gamma$, $\mu$) parameters of the SIRD model. Using these parameters, the differential equations can be solved properly to predict the development trend of the COVID-19 pandemic. The significance of BeCaked model is to make the predictions among these parameters of the differential equations based on the number of infectious, recovered, and deceased cases in the past, instead of accurately predicting the number of those cases. Therefore, it can predict the trend of an increase, decrease, or a peak in the number of susceptible, infectious, recovered and deceased cases. These predictions can help the authorities to give appropriate strategies in order to deal with the spread of this pandemic.

In the evaluation process, we compared the performance between our BeCaked model and current top-tier forecasting-specialized models to prove the reliability of ours. In the global evaluation, our model scored almost the highest performance, while at the country-scale, its effectiveness drops significantly in some countries at 15-step forecast. The mystery behind this unusual is the difference between an open system and a closed system. The global can be considered as a closed system because there is no outside factor affecting the COVID-19 pandemic situation. Contrasting to the global, each country is an open system due to many unforeseen factors such as illegal entry, inaccurate testing, etc. Therefore, when applying any forecasting model to any open system, the most important and effective decision of the model is “how quickly the model is able to catch up with the trend of the pandemic”. Due to that, when comparing at the country level, we only compare up to 15-step forecasting, because the previous study^49^ shows that 14 days is the period for the situation of this pandemic changes.

### Model limitations

Although our model can adapt to the new pandemic situations, it takes time to realize the trend depending on the local policies of the considered area (for example, in the case of Spain, it takes effect immediately while in the case of Russia, it takes about 30 days). This is the foreseen problem because the factors that affect the infection of this pandemic are diverse. Some important factorials such as age, underlying medical conditions, restriction policies, quarantines, etc. are said to be very region-specific and we lack information about those factors. When a new variant of Coronavirus appears, eg. *Delta* or *Omicron*, the performance of our model visibly suffered from less accuracy due to the sudden changes in infected cases.

In practice, when applying machine learning models to forecast an epidemic, forecasting systems often incorporate *reinforcement learning*^57^ strategies to deal with strange situations when involved epidemiological factors change. In our context of COVID-19 prediction, since the pandemic model can always be reflected by the SIRD model with ($\beta$, $\gamma$, $\mu$) parameters, the reinforcement process can help to quickly determine new suitable parameter values once finetuned with recent data. Typically, the operational mechanism of reinforcement learning is as follows.

1. Get model and data for initial training.
2. Train the model with initial data.
3. Do *k*-step forecasting every day with the trained model.
4. If in *m* days, the average difference between the forecast results and the actual number of cases exceeds a certain threshold, the system automatically takes the data of recent days and finetunes the model on those data.
5. In case the difference is within the allowable threshold, the system continues to keep the old model for the next day.

With the above operation flows, the initial trained model can adapt to new situations and produce better up-to-date results. In addition, it is also worth noting that this strategy was really applied with our real system at http://cse.hcmut.edu.vn/BeCaked during the fourth wave of COVID-19 in Ho Chi Minh City, Vietnam under the spreading of the Delta variant at May 2021. Our system caught a sudden change in the real collected data of the city and recent data had been used to finetune the system for a few weeks. The system then became stable again afterward. It showed that using the reinforcement learning strategy, our system can partially solve the problem of sudden changes in data due to unforeseen factors and also helps the model to quickly adapt to the new situation in case of a new variant.

## Conclusion

With the model we have proposed, the pandemic has been modeled and forecasted relatively accurately. We expect this model to be widely applied in each country and region as a reference source in pandemic prevention. In the future, we will continue to experiment with more extensive variations of the SIRD model on more detailed pandemic datasets. Also, we will try to combine many other SOTA techniques for continuously-like data with mathematical models to solve other related problems as mentioned in “Related works”. At the same time, we will also continue to maintain the website, update disease data daily and conduct reinforcement learning methods on the proposed model to have future forecasts as accurate as possible. This model is a non-profit community project, which is of great significance for development in all aspects if properly applied. The forecast of the situation of the COVID-19 pandemic enables the government and citizens together to comply with necessary regulations such as quarantine to prevent the spread of the COVID-19 pandemic. Policies and regulations are essential and most effective when being implemented before it is too late. For example, if we predict the resurgence of the COVID-19 pandemic after a peaceful period, we will be more proactive in preventing as well as reducing the number of people infected and fatal. The COVID-19 pandemic has had a great impact on people’s lives, seriously affecting the economy and society. Therefore, if everyone works together to prevent the epidemic of COVID-19, then socio-economic life can return to stability and continue to develop. This is the core purpose that this study wants to achieve.

Regarding the future work, the combination of VAE, with the classical SIRD model is one of the most interesting and promising medical and computer science projects if it is further developed. The application of computer science, especially Artificial Intelligence in medicine, is a step towards the future. In one day not far, computers can replace humans to do complex things, the things that require constant calculation and repetition. Like forecasting using the SIRD model, computer science in general and Artificial Intelligence in particular play an essential role to approximate the variables $\beta$, $\gamma$ and $\mu$. It is the AI that helps us complete the differential equation to be able to predict the situation of the COVID-19 pandemic. But computer science does not stop at predicting the situation of the COVID-19 pandemic. With this model, we can find the suitable coefficients for problems using the differential equation or other complex stools in issues such as gene sequencing, diet generating, prediction of other diseases, etc. Moreover, with the development of Artificial Intelligence, we can apply it to medicine such as diagnosing, screening disease, revealing risk factors, etc. in each patient. The world is evolving and the intersection of the fields is of utmost importance. This has contributed to making people’s lives become better, especially since human health issues are being cared for more and more. Our explainable AI model of BeCaked still has much room for further improvements. Firstly, the basic SIRD model can be replaced by other upgraded models such as SEIR^7^ or SEIPEHRF^18^. Moreover, we can further encode additional information such as travel history or contact information to make the model predictions closer to practical situations. In terms of Deep Learning techniques, the latest encoding models such as BERT^58^ or GPT-3^59^ can be also considered as well to make the encoded information more meaningful.

## Figures and Tables

**Figure 14:**
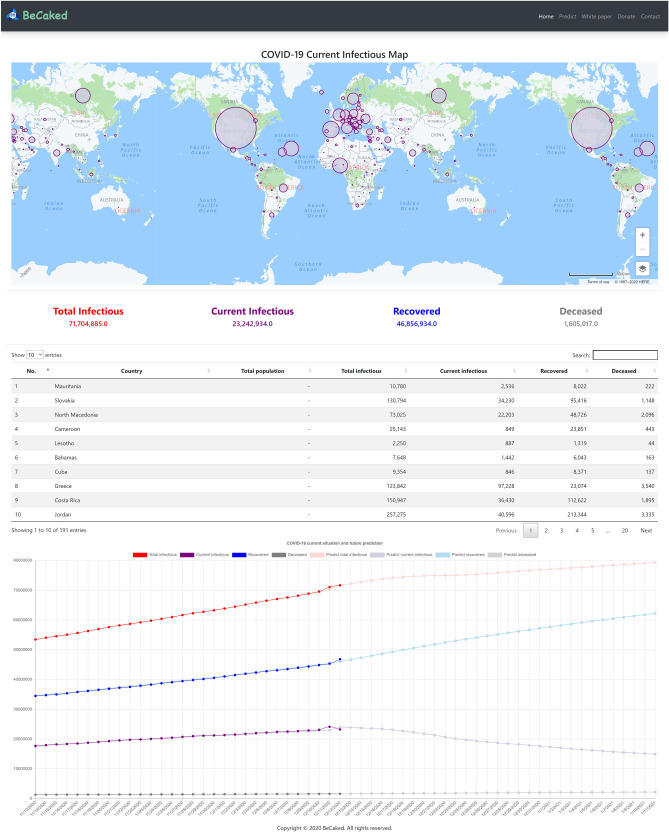
Homepage of the web user interface including COVID-19 map created using HERE Maps^60^.

**Figure 15:**
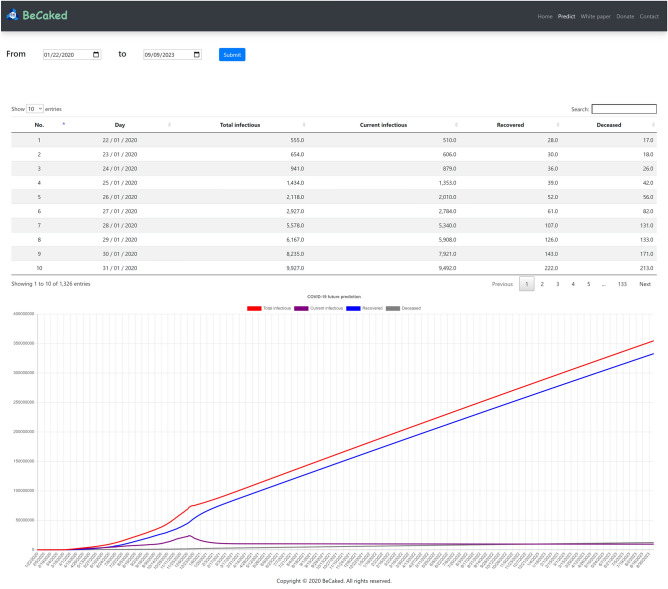
Long-time forecasting page.

## Data Availability

The COVID-19 data analysed during the current study are available from Johns Hopkins University Center for Systems Science and Engineering in the COVID-19 repository, https://github.com/CSSEGISandData/COVID-19. The world and countries population data analysed during the current study are available from Worldometer on their website, https://www.worldometers.info/world-population/population-by-country.

## Appendix 1: The novel name “BeCaked”

In view of the fact that a new variant of coronavirus and COVID-19, the illness they cause, are disastrously spreading among communities in many countries. Therefore, a set of effective non-pharmaceutical interventions, known as social distancing, helps to reduce the spread of this pandemic without pharmaceutical treatments by keeping an acceptable distance from each other (the distance specifically differs from country to country), avoiding gatherings into crowded places. The name “BeCaked” resulting from that strong motivation, standing for Be Careful and Keep Distance, is named in the spirit of encouraging people to strictly comply with the coronavirus legislation in order to protect themselves and their society. Up till now, our website system which can be visited at http://cse.hcmut.edu.vn/BeCaked has fully implemented for visualizing and observing purposes.

## Appendix 2: BeCaked web-based system illustrations

In order to simplify the use of the BeCaked model and respond to a wide variety of users, we implemented a website to statistic and visualize the forecasting results of BeCaked model. Our website includes two main parts: overview and future forecast.

The overview page shown in Fig. 14 includes a map which shows the spread-level of the epidemic in nations, statistics of the latest infectious, recovered and deceased cases, 30 days of epidemic history and a future 30-day forecasting results. Users can change the type of cases shown in the world map by clicking on the total cases of that type located under the map. Also, users can search the cases of a specific country by entering the country name in the search box above the information table.

The forecast for the future shows a long-term forecast for the COVID-19 pandemic. Users can get a reliable prediction for a specific period of time by entering the start and end date. Our system will then return the forecasting results of infectious, recovered and deceased cases of each day, and a line chart represents those metrics to give the user a more overview of the spread of this pandemic during that time. An example of using this page is presented in Fig. 15. In this example, we get the forecasting results from January 22nd 2020 to June 28th 2023. Moreover, users can search for specific day cases by typing the day in the search box.

## Code availability

Our implementation of proposed model is available at https://github.com/nguyenquangduc2000/BeCaked.
